# A Narrative Review of Rotator Cuff Tear Management: Surgery Versus Conservative Treatment

**DOI:** 10.7759/cureus.74988

**Published:** 2024-12-02

**Authors:** Turki Abdulaziz Altamimi, Abdulaziz Ahmed Alkathami, Raghad Mahdi M Al-Awn, Mohammed Hamoud Eid Alkhaldi, Muath Hussain M Alhudaithi, Abdulhadi Ali Alqahtani, Afaf Abdulaziz Salem Alzahrani, Sally Saleh Mohammed Aladwani, Aljalal Fahad Abdulrahman, Ahmed Nasser A Almutawah

**Affiliations:** 1 Orthopedics, King Fahad Medical City, Riyadh, SAU; 2 Orthopedics, Imam Muhammad Ibn Saud Islamic University, Riyadh, SAU; 3 Orthopedics, King Khalid University, Abha, SAU; 4 Orthopedic Surgery, Qurayyat General Hospital, Ministry of Health, Al-Jouf, SAU; 5 Orthopedic Surgery, Aseer Central Hospital, Aseer, SAU; 6 Orthopedic Surgery, King Saud Bin Abdulaziz University for Health Sciences, Riyadh, SAU; 7 Orthopedics, King Khalid University Hospital, Riyadh, SAU; 8 Orthopedics, Princess Nourah Bint Abdulrahman University, Riyadh, SAU; 9 Orthopedics, Imam Muhammad Ibn Saud University, Riyadh, SAU; 10 Orthopedics, King Faisal University, Hofuf, SAU

**Keywords:** arthroscopic repair, conservative management, corticosteroid injections, open repair, platelet-rich plasma therapy, reconstructions, reverse shoulder arthroplasty, rotator cuff tear, surgery, tendon transfers

## Abstract

Rotator cuff tears are a prevalent musculoskeletal issue, particularly among middle-aged and elderly individuals, affecting shoulder stability and arm movement. These tears can arise from acute injuries or chronic wear and tear, leading to conditions ranging from tendinopathy to cuff tear arthropathy. The prevalence increases with age, with a significant portion of older adults affected, many of whom may be asymptomatic. This highlights the necessity for effective management strategies to improve patients' quality of life. The objective of this literature review is to evaluate the evidence on treatment options for rotator cuff injuries, particularly comparing the long-term results of surgical versus non-surgical interventions. While surgical treatments generally provide better functional outcomes and pain relief, non-surgical options like physical therapy and corticosteroid injections can also be effective, especially for smaller tears. The choice of treatment depends on factors like the size of the tear, patient's age, activity level, and overall health. The review highlights the importance of personalized treatment approaches that consider both clinical and economic factors. Although surgical treatments may incur higher initial costs, they often offer superior long-term benefits, especially for younger patients or those with significant tears.

## Introduction and background

Rotator cuff tears (RCTs) are a common musculoskeletal issue, particularly among the middle-aged and the elderly. The rotator cuff is a group of four muscles and their tendons that stabilize the shoulder joint and facilitate various arm movements. Tears can happen due to acute trauma, like a fall or direct impact to the shoulder, or as a result of chronic degenerative changes associated with aging and repetitive overhead activities [1]. Rotator cuff injuries encompass a range of conditions, from tendinopathy and partial-thickness tears to full-thickness tears accompanied by progressive cartilage degeneration, known as cuff tear arthropathy. These injuries can significantly impair shoulder function, leading to pain, weakness, and restricted range of motion (ROM) [2]. 

RCT is the most commonly encountered upper extremity issue among primary-care physicians, physiatrists, and orthopedic specialists [3]. The prevalence of RCTs rises with age, impacting approximately 30% of individuals in their 60s and more than 60% of those over 80 years old [4]. Screening studies utilizing shoulder ultrasonography have provided a clearer understanding of the prevalence of RCTs. For instance, a cross-sectional study conducted in the UK with 1,000 women revealed that 22.2% had full-thickness RCTs. The study also found a positive correlation between the presence of tears and factors such as advancing age and involvement of the dominant shoulder [5]. Similarly, a prospective study conducted in Germany involving 411 asymptomatic shoulders revealed that RCTs were present in 23% of the cases. The prevalence increased with advancing in age, affecting 31% of individuals aged 70 and 51% of those aged 80 years [6]. The incidence of symptomatic RCTs is estimated to be around 2% per year in the general population. However, many tears are asymptomatic and may go undiagnosed, particularly in older adults [5].

**Figure 1:**
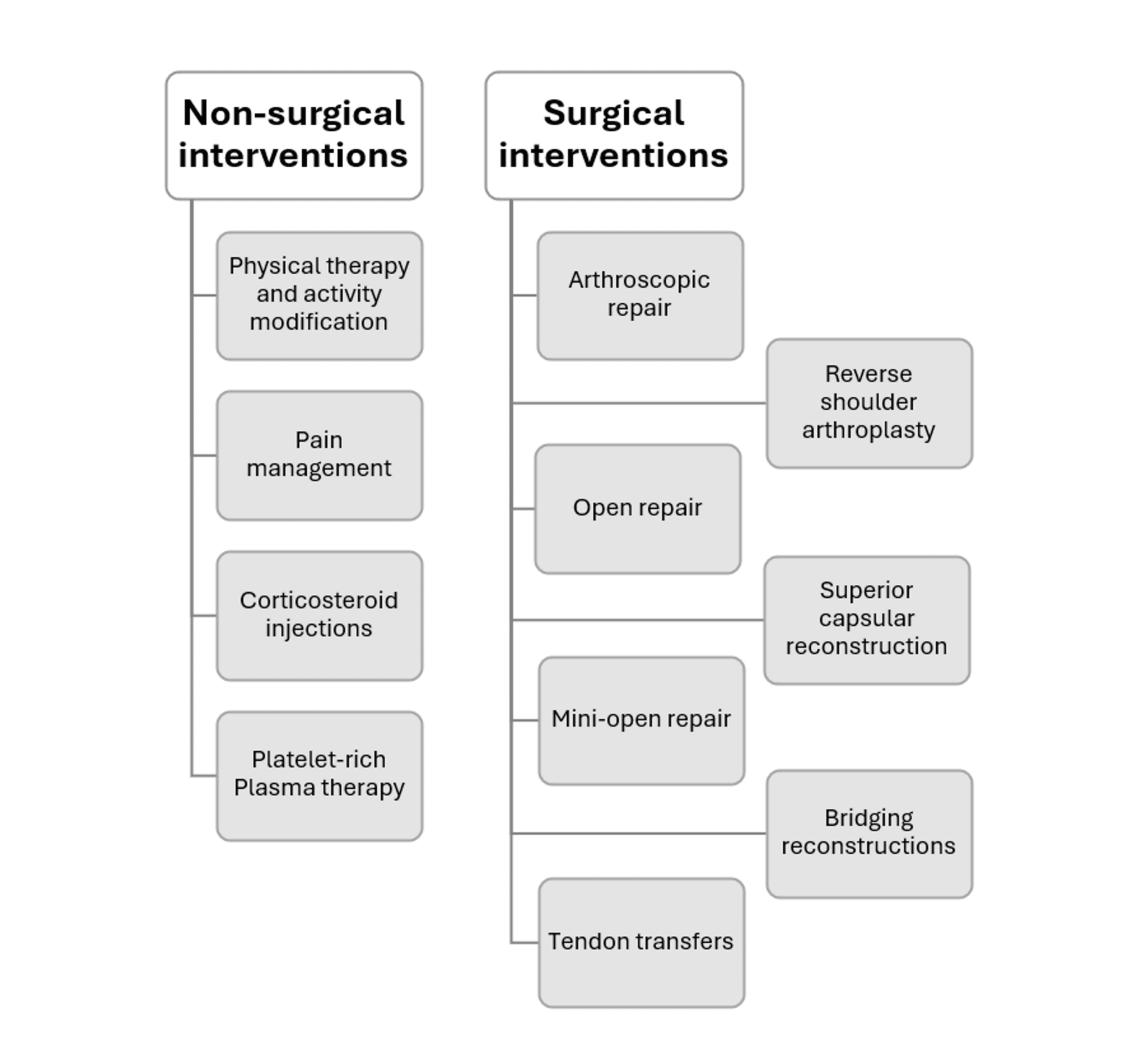
Different surgical and non-surgical interventions for the management of RCTs. This figure is original and created by the authors. RCT: Rotator cuff tears

The high prevalence and incidence of these injuries underscore the importance of effective management strategies to enhance patient outcomes and overall quality of life (QoL). Despite the high prevalence of RCTs, there is ongoing debate about the best management approach. Surgical interventions are commonly performed to restore shoulder function and alleviate pain. However, non-surgical treatments, including physical therapy (PT) and corticosteroid injections, are also widely used and can be effective for certain patients (Figure 1) [7,8]. The choice between surgical and non-surgical management is influenced by several factors, such as the tear’s size and location, the patient’s age, activity level, and comorbidities [9]. For instance, a study by Liu et al. concluded that early surgical repair of traumatic RCTs resulted in better functional outcomes compared to delayed repair [7]. However, another study indicated no notable difference in patient-reported outcomes between surgical and non-surgical treatments for small and medium-sized tears [10].

There is a significant gap in knowledge regarding the long-term outcomes of surgical versus non-surgical interventions for RCTs. While some studies suggest that surgical repair provides superior functional outcomes and pain relief, others indicate that non-surgical treatments can achieve comparable results, particularly for small and medium-sized tears [7,8]. This review aims to summarize and evaluate the existing evidence on the management of RCTs and compare the short-term and long-term outcomes of different treatment modalities.

## Review

Methods

In October 2024, a comprehensive search was performed across PubMed, Scopus, Cochrane, and Web of Science using the keywords “Rotator cuff,” “Tear,” “Surgical management,” and “Non-surgical management,” using Medical Subject Headings (MeSH). Key references were also identified from the bibliographies of relevant studies. We did not apply any filters or limitations during the search process, except for focusing on studies published between 2014 and 2024.

Structure and function of the rotator cuff

The rotator cuff is a complex anatomical structure comprising four key muscles: the supraspinatus, infraspinatus, teres minor, and subscapularis. These originate from the scapula and insert into the humerus, forming a cuff around the glenohumeral joint. This configuration is crucial for the stability and mobility of the shoulder, allowing for a wide range of movements such as abduction, rotation, and elevation of the arm [11]. Functionally, these muscles work synergistically to stabilize the humeral head within the shallow glenoid cavity of the scapula. This stabilization is essential during dynamic activities, preventing dislocation and ensuring smooth articulation of the shoulder joint. The supraspinatus muscle initiates abduction, while the infraspinatus and teres minor facilitate external rotation. The subscapularis, the largest of the four, is responsible for internal rotation [12].

Pathophysiology and etiology of RCTs

The pathophysiology of RCTs involves intrinsic and extrinsic factors. The intrinsic factors include age-related degeneration, reduced vascularity, and tendon attrition, while extrinsic factors encompass mechanical impingement and trauma [13]. Age-related degeneration is a primary intrinsic factor contributing to RCTs. As individuals age, the tendons undergo mucoid degeneration, characterized by the accumulation of glycosaminoglycans and a decrease in collagen organization. This degeneration weakens the tendons, making them more susceptible to tears [14]. Additionally, the vascular supply to the cuff tendons diminishes with age, impairing the tendons’ ability to repair and maintain their structural integrity. These tears are more frequently observed in individuals older than 40 [15].

Extrinsic factors, such as mechanical impingement, also have a significant role in the occurrence of RCTs. Repetitive overhead activities, common in athletes and certain occupations, can result in subacromial impingement. This repetitive compression causes microtrauma and subsequent tendon degeneration. Acute trauma, such as falls or heavy lifting, can also result in RCTs, particularly in younger individuals [16].

Clinical presentation and diagnosis of RCTs

RCTs are a leading cause of shoulder pain and dysfunction, particularly among older adults and athletes. The presentation of RCTs can vary widely, ranging from asymptomatic patients to severe pain and functional impairment. Common symptoms include shoulder pain, especially when lifting the arm, weakness, and a limited ROM. Patients often report difficulty performing overhead activities and may experience night pain that disrupts sleep. In acute cases, such as those resulting from trauma, patients might feel a sudden tearing sensation followed by immediate pain and weakness [17].

The clinical examination of a patient with a suspected RCT involves a thorough assessment of shoulder function and strength. Key physical examination tests include the Neer and Hawkins-Kennedy impingement signs, which help identify subacromial impingement, a common precursor to RCTs. Specific tests for rotator cuff integrity include the drop arm test, the empty can test, and the external rotation lag sign. These tests evaluate the function of the supraspinatus, infraspinatus, and teres minor muscles, respectively. A positive result in these tests, characterized by pain or weakness, strongly suggests a RCT [18,19].

Accurate diagnosis of RCTs often requires imaging studies to confirm clinical findings and to plan appropriate management. MRI is regarded as the method of choice in the diagnosis of RCTs due to its high sensitivity and specificity. MRI offers detailed imaging of shoulder soft tissues and bones, enabling the evaluation of tear size, location, and severity. It also identifies related conditions like muscle atrophy, fatty infiltration, and joint abnormalities, making it essential for preoperative planning and assessing surgical repair prognosis [20].

Ultrasound is a cost-effective modality that is increasingly used in the diagnosis of RCTs. It allows for real-time evaluation of the rotator cuff tendons and is also used to guide therapeutic interventions such as injections. Additionally, ultrasound can be used to assess tendon healing post-operatively and to monitor the progression of conservative treatment [17,21].

While X-rays do not directly visualize RCTs, they are useful in identifying bony abnormalities that may be associated with rotator cuff pathology. These include acromial spurs, which can contribute to subacromial impingement and calcific deposits within the tendons. X-rays can also reveal joint space narrowing and osteophyte formation, which may indicate concurrent osteoarthritis [22].

Non-surgical interventions

Management of RCTs involves both surgical and non-surgical approaches. The choice of treatment depends on the tear severity, age, and activity level of the patient [23]. Non-surgical options are often the first line of management, particularly for partial tears or patients who are not suitable candidates for surgery. These interventions aim to alleviate pain and improve function and the QoL.

PT and Activity Modification

PT is a cornerstone of non-surgical treatment for RCTs. PT programs typically include exercises to improve ROM, strengthen the shoulder muscles, and improve overall shoulder function. Kuhn et al. found that PT effectively reduces pain and enhances function in patients with RCTs [24]. More recent studies have supported these findings, emphasizing the importance of individualized PT programs tailored to the patient’s specific needs and tear severity [25,26].

Activity modification involves altering daily activities to avoid movements that exacerbate symptoms. This can include avoiding overhead activities, heavy lifting, and repetitive shoulder movements. Activity modification, combined with other non-surgical treatments, can significantly reduce pain and improve shoulder function. Patients are often advised to gradually reintroduce activities as their symptoms improve [27].

Pain Management 

Effective pain management is essential in the non-surgical management of RCTs. Non-steroidal anti-inflammatory drugs (NSAIDs) are commonly used to reduce inflammation and alleviate pain. For patients who cannot tolerate NSAIDs, acetaminophen serves as a viable alternative [28].

Additionally, alternative options like acupuncture, transcutaneous electrical nerve stimulation (TENS), and ultrasound therapy have shown promise in managing pain. Mahure et al. found that TENS significantly reduced pain intensity by 30% in 70% of participants, highlighting its efficacy as a pain management tool [28,29].

Corticosteroid Injections

Corticosteroid injections are commonly used for the treatment of RCTs due to their potent anti-inflammatory effects. In the short term, these injections can significantly reduce pain and improve function. Studies have shown that patients often experience relief within a few days to weeks post-injection, with notable improvements in pain scores and ROM. This immediate relief can facilitate participation in PT and other rehabilitative exercises, potentially enhancing overall recovery [30]. The long-term efficacy of corticosteroid injections for RCTs is more contentious. While short-term benefits are well-documented, the long-term outcomes are less favorable. Research indicates that the initial pain relief may not be sustained beyond a few months, and repeated injections can lead to diminishing returns [31]. 

Several factors can influence the effectiveness of corticosteroid injections. The severity and chronicity of the RCT, the specific corticosteroid used, and the injection technique, all play critical roles. Patient-related factors like age, activity level, and comorbidities (e.g., diabetes) can also impact outcomes [32]. For instance, patients with chronic tears or those who delay treatment may experience less pronounced benefits compared to those with acute injuries [33]. Common side effects include post-injection flare (transient increase in pain), skin discoloration, and subcutaneous fat atrophy at the injection site. More serious complications, though rare, include infection and tendon rupture [34]. Additionally, there is evidence indicating that frequent corticosteroid injections may have a role in tendon degeneration and weakening, potentially exacerbating the underlying condition [35].

Platelet-Rich Plasma Therapy

Platelet-rich plasma (PRP) therapy has emerged as a promising non-surgical intervention for RCTs. Short-term outcomes of PRP therapy often include substantial pain relief and enhanced shoulder function within the first six months post treatment. PRP therapy can enhance the healing process by promoting tissue regeneration and reducing inflammation [36,37]. Even so, the long-term outcomes are more variable. Some patients maintain improved function and reduced pain for up to two years, while others may experience a decline in benefits over time [38].

Several factors can influence the outcomes of PRP therapy, including the tear severity, the age of the patient, and the specific PRP preparation used. For instance, a network meta-analysis conducted in 2020 concluded that higher concentrations of platelets and leukocytes in the PRP preparation are associated with a reduced rate of retear and deficient tendon healing, as well as improved patient-reported outcomes [39]. Despite its benefits, common adverse effects include pain at the injection site, swelling, and, in rare cases, infection. These side effects are generally minor and self-limiting [37].

Surgical interventions 

Arthroscopic Repair

Arthroscopic repair is a widely utilized surgical intervention for RCTs. It is preferred due to its minimally invasive approach, which offers advantages like less post-operative pain and quicker recovery periods. This technique includes using small incisions and an arthroscope to visualize and repair the damaged tendons. In the short term, arthroscopic repair has demonstrated a notable improvement in shoulder function and alleviation of pain [40]. Patients typically experience notable improvements in pain scores, including the Visual Analog Scale (VAS), and functional scores, including the American Shoulder and Elbow Surgeons (ASES) score and the Constant-Murley score (CMS), within the first six months post surgery [41,42]. Liu et al. concluded that early arthroscopic repair of traumatic RCTs resulted in better outcomes, including enhanced ROM and lower pain symptoms, compared to delayed repair. Additionally, patients who received early repair experienced a reduced risk of post-operative re-tears [7].

The long-term results of arthroscopic repair are typically favorable, with many patients achieving sustained improvements in shoulder function and pain reduction. The benefits of arthroscopic repair can persist for several years post-surgery [43]. For instance, a network meta-analysis by You et al. reported that arthroscopic repair techniques, including single-row and double-row repairs, provided notable long-term improvements in functional scores and pain relief [44]. However, the risk of re-tear continues to be a concern, especially among older patients and those with larger tears [42].

Factors influencing the results of arthroscopic RCT repair include patient age, tear size, chronicity, tendon quality, and comorbidities. Older patients tend to have higher re-tear rates and may experience less favorable outcomes compared to younger patients [42]. Larger and chronic tears are associated with poorer outcomes and higher re-tear rates. Poor tendon quality, often due to degeneration, can negatively impact the success of the repair. Additionally, conditions like diabetes and smoking can negatively impact healing and increase the possibility of complications [1,45].

Despite its benefits, arthroscopic repair has some risks. Common complications include re-tear, infection, stiffness, and nerve injury. Re-tear is the most significant complication, with rates varying based on patient and tear characteristics [46]. Although rare, infections can occur and may require additional treatment. Post-operative stiffness is a common issue, often managed with PT. There is a minor risk of nerve injury associated with the procedure, which can lead to temporary or permanent deficits [47]. In conclusion, arthroscopic repair is a highly effective surgical intervention for RCTs, offering significant short-term and long-term benefits. However, outcomes can be influenced by various factors, and potential complications must be considered.

Open Repair 

Open repair is a traditional surgical intervention for RCTs, particularly indicated for large or complex tears that may not be amenable to arthroscopic techniques. This procedure involves a larger incision to directly visualize and repair the torn tendons, allowing for a more comprehensive approach to tendon reattachment and reconstruction [48]. Open repair has been extensively studied for its efficacy and outcomes. In the short term, open repair typically leads to significant pain relief and improvement in the function of the shoulder, with many patients experiencing substantial gains in strength and ROM within the first six months post surgery. However, the recovery period can be longer compared to arthroscopic techniques, often requiring a more extended rehabilitation phase [9]. A non-randomized controlled trial comparing the outcomes of open repair and arthroscopic repair for full-thickness RCTs concluded that the outcomes of open repair for small-to-massive tears were comparable to those of arthroscopic repair. However, patients with large-to-massive tears had poorer outcomes compared to those with small-to-medium tears, irrespective of the repair method used [49]. Long-term results of open repair are generally positive, with studies reporting sustained improvements in the functionality of the shoulder and patient satisfaction up to ten years post-operatively [50]. 

Common complications linked to open repair involve infections, joint stiffness, and the recurrence of tendon tears. A study comparing the short-term complications of RCTs repair using open and arthroscopic techniques found that total complications were more following open repair. Risk factors for complications were identified as being over 65 years old, having an operative time longer than 90 minutes, and undergoing open repair. Open repair was notably linked to a higher likelihood of surgical infections. The higher risk of infection in open repair is attributed to the larger incision required, which can also result in more significant scarring and longer healing times [47]. Overall, while open repair remains an available option for managing RCTs, particularly in cases where arthroscopic repair is not feasible, it is important to weigh the possible benefits against the risks and complications in this procedure.

Mini-Open Repair

Mini-open repair is a hybrid surgical technique for RCTs that combines elements of both open and arthroscopic methods. This approach involves an initial arthroscopic evaluation and preparation of the tear, followed by a smaller incision than traditional open repair to complete the tendon reattachment. The mini-open technique aims to balance the benefits of both methods, offering the direct visualization and access of open repair while minimizing the invasiveness associated with larger incisions [51]. 

A clinical trial comparing the outcomes of all-arthroscopic and mini-open repair of RCTs concluded that the all-arthroscopic approach resulted in less pain, lower disabilities of the arm, shoulder and hand (DASH) scores, and higher CMS during the early recovery period. However, both groups showed no notable differences in long-term primary and secondary outcomes, or in the rates of complications like adhesive capsulitis and rotator cuff re-tear [52]. Also, a meta-analysis of 21 studies encompassing 1,644 surgeries found that arthroscopic and mini-open repairs resulted in comparable outcomes. The analysis revealed that being male and older in age are linked to higher rates of rotator cuff re-tears, while longer surgical procedures are associated with an increased incidence of adhesive capsulitis [53]. 

Tendon Transfers

Tendon transfers are used particularly in cases where the tendon quality is poor or the tear is too extensive to be repaired directly. This procedure involves rerouting a healthy tendon from a different muscle to substitute the function of the damaged rotator cuff tendon. Frequently utilized tendons for transfer are the latissimus dorsi, pectoralis major, and trapezius [54]. The latissimus dorsi, either alone or in combination with the teres major, is frequently used for treating irreparable posterosuperior tears [55]. The latissimus dorsi is an excellent choice for muscle transfer procedures due to its extensive muscle excursion [56]. 

A systematic review conducted in 2012 assessed the results, predictive factors, and complications of latissimus dorsi transfers. The review, with an average follow-up period of 45.5 months, identified substantial post-operative enhancements in the CMS, active forward elevation, abduction, and external rotation. However, the complication rate was 9.5%, including issues like infection and tendon tears [57]. Another study examined the impact of including a small bone fragment with the latissimus dorsi during tendon transfer compared to sharp tendon separation from the humerus. Its findings indicated that the group with the tendon harvested along with a bone chip reported significantly better outcomes in terms of ASES score, mean CMS, ROM, and strength [56]. Another recent systematic review conducted in 2024 concluded that latissimus dorsi tendon transfer greatly enhances patient-reported outcomes, alleviates pain, and improves ROM and strength, while maintaining low rates of complications and revision surgeries over mid- to long-term follow-up periods [58].

Reverse Shoulder Arthroplasty

Reverse shoulder arthroplasty (RSA) is regarded as a major technological breakthrough in shoulder reconstructive surgery over the last three decades. It effectively reduces pain and enhances function in patients with RCTs [59]. This technique reshapes the glenoid into a spherical form to engage with a humeral socket. The contemporary reverse prosthesis repositions the center of rotation both medially and distally, which boosts the deltoid muscle’s mechanical advantage. Consequently, this design has markedly enhanced active shoulder elevation and overall QoL [60].

RSA is effective and safe, with patients showing significant enhancements in ROM and outcome scores. The procedure has a low overall complications rate of 9.4% and a revision rate of just 2.6%, with the most common complications being scapular notching, periprosthetic fractures, glenoid loosening, and prosthetic dislocation [61]. Patients with massive RCTs who do not have osteoarthritis are more likely to achieve pain relief and functional enhancements after undergoing RSA [62]. 

Fatty infiltration in the rotator cuff muscles, particularly the teres minor and infraspinatus, before surgery negatively impacts patients’ perceived outcomes and the recovery of ROM, especially external rotation, after RSA [63].

Superior Capsular Reconstruction

Superior capsular reconstruction (SCR) was initially introduced by Japanese surgeon Teruhisa Mihata in 2012, at a time when RSA implants were not available to him. This technique quickly gained acceptance and became widely utilized within the orthopedic community [64]. 

Recent studies indicate that SCR can yield favorable short-term outcomes, including pain reduction and improved shoulder function. A meta-analysis concluded that SCR significantly improves the ROM and functional scores in patients, with notable gains in abduction, elevation, external rotation, and internal rotation. VAS, CMS, subjective shoulder value (SSV), and ASES, also showed significant improvements. The healing rate was 76.1%, with a complication rate of 5.6% and a RSA revision rate of 7.1% [65].

Bridging Reconstructions

Bridging reconstruction, an early arthroscopic method for addressing irreparable RCTs continues to be a valuable technique in shoulder surgery [66]. This technique is especially effective for treating irreparable tears in the superior-posterior rotator cuff when the subscapularis is intact or can be repaired, particularly in patients who have maintained good preoperative function and ROM. Historically, the majority of research on bridging reconstruction has been derived from case series conducted at individual institutions [67-69]. However, a notable study by Karpyshyn et al. compared the healing outcomes of bridging reconstruction and interpositional dermal allograft to maximal repair. The study revealed that the first group showed a higher healing rate and a tendency for better preservation of supraspinatus muscle mass in comparison to the maximal repair group. This suggests that lower-tension allografts may heal more effectively in comparison to higher-tension native tendons, particularly in cases where the maximal repair only achieved partial repair due to the irreparable nature of the tears. Although the sample size was limited to 30 patients, these findings reinforce the viability of bridging reconstruction as a treatment option for irreparable RCTs [70].

Comparative analysis of surgical vs non-surgical interventions 

The treatment of RCTs involves a critical decision between surgical and non-surgical options, each with distinct outcomes and implications. Schmucker et al. compared the efficacy and safety of these approaches for full-thickness RCTs. The review included ten studies, comprising both randomized and non-randomized clinical trials, and found that surgical treatment generally led to superior outcomes concerning shoulder function and pain reduction. Specifically, one year post treatment, patients who underwent surgery showed a notable improvement in the CMS and an alleviation of pain measured by the VAS. These benefits persisted in some cases up to 10 years, suggesting long-term advantages of surgical intervention. However, the review also noted that for other outcomes such as ROM, muscle strength, QoL, and adverse events, the differences between surgical and non-surgical treatments were less pronounced [71].

In contrast, Fahy et al. focused on the effectiveness of exercise compared to surgery for large to massive RCTs. This study highlighted that exercise interventions could be comparable in effectiveness with surgical treatments in improving QoL, disability, and pain. The analysis included five trials with a total of 297 participants and found that, at 12 months, exercise led to significant improvements in shoulder external rotation ROM, while surgery showed a slight advantage in pain reduction. The study emphasized the importance of high-quality exercise programs and noted significant discrepancies in the research on exercise interventions in the literature. Despite these variations, the findings indicate that non-surgical interventions, particularly structured exercise programs, can provide significant benefits for patients with large to massive RCTs, offering a viable alternative to surgery. However, it is important to note that this conclusion is drawn from evidence of low certainty [72].

Overall, the comparative analysis reveals that while surgical treatment may offer superior clinical outcomes and pain relief in the short and long term, non-surgical approaches, especially exercise, can also lead to notable improvements in patient-reported outcomes. The choice between these interventions should consider the severity of the tear, patient comorbidities, and individual preferences, with a tailored approach to improve clinical outcomes and QoL.

Comparative analysis of multiple surgical interventions

A comparative analysis of multiple surgical interventions for RCTs reveals significant insights into their efficacy and outcomes. The study by Zhou et al. systematically reviewed the outcomes of various surgical techniques, including arthroscopic repair, open repair, and SCR. The findings indicated that arthroscopic repair generally yielded better functional outcomes and lower complication rates compared to open repair. Specifically, patients undergoing arthroscopic repair demonstrated significant improvements in shoulder function and pain relief, with fewer incidences of post-operative complications such as infection and deltoid detachment. In contrast, open repair, while effective in certain cases, was linked to higher risks of complications and longer recovery periods. This underscores the importance of selecting the appropriate surgical technique based on the condition of the patient and the expertise of the surgeon [73].

Further, a network meta-analysis by Bi et al. provides a comprehensive evaluation of various surgical interventions. The findings indicate that arthroscopic bridging graft and SCR yield the highest improvements in ASES scores. Debridement and arthroscopic bridging graft show significant benefits in CMS, while SCR excels in enhancing acromiohumeral distance [74]. Additionally, Kovacevic et al. examined the management of irreparable massive RCTs, comparing patient-reported outcomes, reoperation rates, and treatment responses across different surgical strategies. The analysis revealed that SCR and tendon transfer techniques provided significant improvements in patient-reported outcomes, like the CMS and the ASES score. However, these techniques also had varying rates of complications and reoperations, with SCR showing a relatively lower reoperation rate compared to tendon transfer [75].

In conclusion, the comparative analysis highlights the nuanced differences in outcomes and complications associated with each technique. Arthroscopic repair generally offers better functional outcomes and lower complication rates, while bridging reconstruction and SCR provide viable alternatives for managing large to massive tears with specific advantages in structural integrity and patient-reported outcomes. These findings highlight the significance of personalized treatment approaches to improve surgical outcomes and enhance patient QoL.

Evaluating the cost-effectiveness of various treatment strategies

Nicholson et al. conducted a comparative analysis of the cost-effectiveness of surgical interventions and non-surgical management for RCTs, focusing on the economic and patient satisfaction outcomes of arthroscopic repair. Their findings indicate that arthroscopic repair is generally cost-effective, particularly for younger patients, due to significant improvements in functional scores and quality-adjusted life years (QALYs). The incremental cost-effectiveness ratio (ICER) for arthroscopic repair is favorable, especially when considering the long-term benefits and the reduced need for subsequent interventions [76].

Also, Kang et al. examined various surgical options, including SCR, tendon transfer, and RSA, alongside non-surgical management. Their study found that while non-surgical management is the least costly, it often results in lower functional outcomes and higher long-term healthcare costs due to ongoing treatment needs. Among the surgical options, SCR and RSA, despite their higher initial costs, provide better functional outcomes and higher QALYs, making them cost-effective in the long run. The ICERs for SCR and RSA fall within acceptable thresholds, indicating their economic viability for patients with irreparable tears [77].

Rehabilitation and post-treatment care

Rehabilitation and post-treatment care are critical components in the management of RCTs, whether treated surgically or non-surgically. The primary goals of rehabilitation include pain reduction, restoration of function, and prevention of further injury. For non-surgical management, rehabilitation typically begins with a focus on pain control and inflammation reduction [78]. Early stages emphasize passive ROM exercises to maintain joint mobility without stressing the healing tissues. As pain decreases, active-assisted and active ROM exercises are introduced to enhance flexibility and strength. Strengthening exercises, particularly those targeting the rotator cuff and scapular stabilizers, are gradually incorporated to improve shoulder stability and function [79].

In the context of surgical repair, rehabilitation protocols are more structured and phased. Initially, the focus is on protecting the surgical repair, minimizing pain and swelling, and maintaining ROM in adjacent joints. Patients are often immobilized in a sling for several weeks, with passive ROM exercises commencing early to prevent stiffness. As healing progresses, active-assisted and active ROM exercises are introduced, followed by gradual strengthening exercises [80]. The progression through these phases is carefully monitored to avoid overloading the repaired tendon. Long-term rehabilitation aims to restore full function and strength, with a focus on functional exercises that mimic daily activities and sports-specific movements [80]. A systematic review conducted in 2021 found that supervised PT and bracing in 15 degrees external rotation after arthroscopic repair are effective. Early isometric loading showed improved outcomes in one study, while TENS helped manage pain [81]. Another systematic review by Bandara et al. compares conservative and aggressive rehabilitation protocols post-surgery. They found no clear benefit in ROM outcomes but the findings suggest a slight functional improvement with aggressive rehabilitation [82]. Overall, a well-structured rehabilitation program is vital for achieving optimal outcomes in the management of RCTs, regardless of the treatment modality chosen.

Implications for clinical practice and current trends and future directions

The management of RCTs has significant implications for clinical practice, particularly in tailoring treatment approaches to individual patient needs. Clinicians must consider factors such as tear size, patient age, activity level, and overall health when deciding between surgical and non-surgical interventions. Surgical options, including arthroscopic repair, open repair, SCR, tendon transfers, and RSA, offer varying benefits and risks. Non-surgical management, often involving PT and corticosteroid injections, remains a viable option for patients with smaller tears or those who are not surgical candidates. Current trends in the field highlight advances in surgical approaches, such as the development of minimally invasive procedures and the use of biologic augmentation to enhance healing [83]. Innovations like SCR and tendon transfer have expanded the surgical repertoire, providing solutions for massive, irreparable tears that were previously deemed untreatable. Additionally, there is an increasing interest in the use of regenerative medicine, including PRP therapy and stem cell therapy, to promote tissue repair and improve outcomes in non-surgical management [84]. Future research directions are focused on optimizing patient selection criteria, improving surgical techniques, and developing novel non-surgical treatments. Furthermore, advancements in imaging technology and biomechanical modeling are expected to enhance preoperative planning and post-operative rehabilitation protocols.

## Conclusions

The comparison of surgical and non-surgical interventions for RCTs highlights the nuanced benefits and limitations of each approach. While surgical options, particularly arthroscopic repair, generally yield superior functional outcomes and pain relief, structured non-surgical interventions, such as PT and activity modifications, also demonstrate notable improvements in patient-reported outcomes. These findings underscore the necessity of personalized treatment strategies that consider the severity of the tear, patient comorbidities, and individual preferences to optimize clinical outcomes.

Furthermore, the evaluation of cost-effectiveness reinforces the importance of selecting appropriate interventions based on both clinical and economic factors. Surgical treatments, despite their higher initial costs, tend to offer better long-term benefits and QALYs, particularly for younger patients and those with massive irreparable tears. Ultimately, a comprehensive understanding of the efficacy, safety, and economic implications of each treatment strategy is essential for guiding clinical decision-making and enhancing patient QoL.
